# Evaluation of Anticholinesterase Activity of the Fungicides Mefentrifluconazole and Pyraclostrobin

**DOI:** 10.3390/ijms25126310

**Published:** 2024-06-07

**Authors:** Dora Kolić, Goran Šinko

**Affiliations:** Institute for Medical Research and Occupational Health, Ksaverska cesta 2, HR-10000 Zagreb, Croatia; dkolic@imi.hr

**Keywords:** fungicide, cholinesterase, triazole, carbamate, molecular docking

## Abstract

Triazoles are compounds with various biological activities, including fungicidal action. They became popular through cholinesterase studies after the successful synthesis of the dual binding femtomolar triazole inhibitor of acetylcholinesterase (AChE, EC 3.1.1.7) by Sharpless et al. via *in situ* click chemistry. Here, we evaluate the anticholinesterase effect of the first isopropanol triazole fungicide mefentrifluconazole (Ravystar^®^), developed to overcome fungus resistance in plant disease management. Mefentrifluconazole is commercially available individually or in a binary fungicidal mixture, i.e., with pyraclostrobin (Ravycare^®^). Pyraclostrobin is a carbamate that contains a pyrazole ring. Carbamates are known inhibitors of cholinesterases and the carbamate rivastigmine is already in use for the treatment of Alzheimer’s disease. We tested the type and potency of anticholinesterase activity of mefentrifluconazole and pyraclostrobin. Mefentrifluconazole reversibly inhibited human AChE and BChE with a seven-fold higher potency toward AChE (*K*_i_ = 101 ± 19 μM). Pyraclostrobin (50 μM) inhibited AChE and BChE progressively with rate constants of (t_1/2_ = 2.1 min; *k*_i_ = 6.6 × 10^3^ M^−1^ min^−1^) and (t_1/2_ = 1.5 min; *k*_i_ = 9.2 × 10^3^ M^−1^ min^−1^), respectively. A molecular docking study indicated key interactions between the tested fungicides and residues of the lipophilic active site of AChE and BChE. Additionally, the physicochemical properties of the tested fungicides were compared to values for CNS-active drugs to estimate the blood–brain barrier permeability. Our results can be applied in the design of new molecules with a lesser impact on humans and the environment.

## 1. Introduction

**Figure 1:**
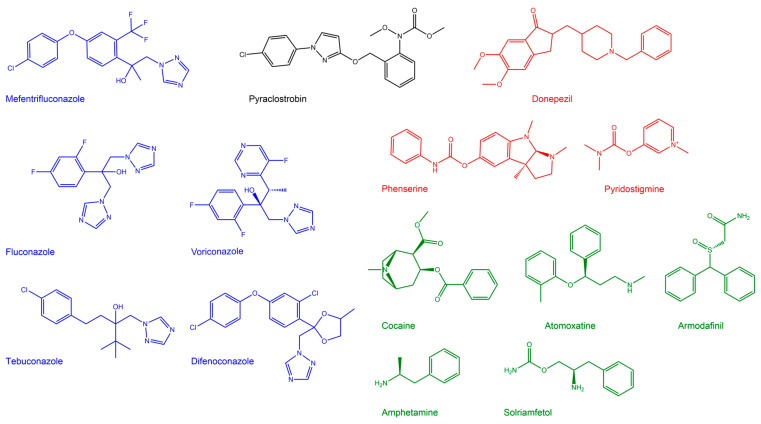
Molecular structure of triazole compounds (blue), anticholinesterase compounds (red) and central nervous stimulants (green). For all of the molecular structures used in this study, please see Figure S1.

Modern agronomy is based on plant disease management with the goal of reducing crop loss due to fungal diseases [1]. Azole drugs emerged in the 1960s as inhibitors of fungal cytochrome CYP51 family enzymes, preventing the conversion of lanosterol to ergosterol, which is a regulator of membrane fluidity and permeability [2]. Azoles are currently the most used class of antifungals in agriculture and medicine [2,3]. There are two main groups of antifungal drugs; imidazole- and triazole-based. Besides crop protection, antifungal drugs are also used in human medicine known as antimycotic drugs, i.e., imidazoles clotrimazole, econazole, and miconazole, while fluconazole, ketoconazole and voriconazole fall under the triazole class (Figure 1) [2,3]. Unwanted cross-reactivity with human cytochrome P450s enzymes forced the development of safer azoles, and it was shown that voriconazole inhibited fewer human P450s [3,4]. Voriconazole is a drug that was created by replacing one of two fluconazole’s azole groups with a fluoropyrimidine group. It is characterized by good adsorption property regarding lipophilicity and acceptable hepatotoxicity [5].

Antifungal resistance represents a serious threat in crop production and has led to the development of a novel triazole fungicide–mefentrifluconazole, the first isopropanol triazole fungicide, which, like voriconazole, inhibited fewer human P450s, thus being among the safer azoles [6,7,8]. Mefentrifluconazole selectivity for fungal cytochrome (*Zymoseptoria tritici* CYP51; *K*_D_ = 0.5 nM) in comparison to human aromatase cytochrome (CYP19; *K*_D_ = 920 nM) is about 1800-fold, making it a safer azole for humans due to its high selectivity. Interestingly, an unwanted side effect of aromatase inhibition has been exploited in the development of azole derivatives to treat estrogen-dependent breast cancer [9], with fadrozole having an IC_50_ of 0.0076 μM in human aromatase supersomes [10].

Although the primary concern about azole toxicity is attributed to human cytochrome selectivity, azole drugs also have other unwanted side effects. In the central nervous system (CNS), adverse effects of the triazole fungicide drug fluconazole overlap with adverse effects caused by anticholinergic drugs including dizziness, tremors, convulsions, etc. [11]. This fact brings to attention the need to determine the effect of triazole fungicides on cholinesterase activity, acetylcholinesterase (AChE; EC 3.1.1.7) and the related butyrylcholinesterase (BChE; EC 3.1.1.8).

AChE is an essential enzyme for cholinergic neurotransmission [2,3]. Severe inhibition may even cause death via apnea in case of organophosphorus compounds poisoning, i.e., nerve agents. Organophosphorus compounds used as pest control agents account for numerous cases of occupational hazards and suicide attempts mainly in developing countries [12]. The related enzyme BChE, whose metabolic role is not essential, may serve as a CNS co-regulator of cholinergic neurotransmission [6], and therefore BChE inhibition coupled with AChE inhibition may harm a healthy person.

Individuals suffering from Alzheimer’s disease (AD) use current therapy targeting acetylcholine levels in CNS synapses by applying selective AChE inhibitors: donepezil, rivastigmine and galantamine. Carbamate rivastigmine inhibits both AChE and BChE, unlike donepezil and galantamine, which selectively inhibit AChE. Since during AD progression BChE increases up to approx. two-fold of normal activity in some brain regions, selective BChE inhibitors may act as therapeutics for later AD stages [13,14]. Analyzing the CNS-active drug scaffold and comparing it to other CNS-active compounds may be used to increase the safety of unwanted effects or increase therapeutic effects for targeted biomolecules. In addition, AD is also targeted using antibody therapy under the amyloid hypothesis for AD [15,16].

Triazoles became popular through cholinesterase studies after the successful synthesis of the dual binding femtomolar 1,2,3-triazole inhibitor of AChE by Sharpless et al. via in situ click chemistry using the active site of *Electrophorus* AChE as a reaction vessel [17]. Synthesis of 1,2,3-triazoles via click chemistry using azide–alkyne cycloaddition [18,19,20] enabled the design of novel potent inhibitors of AChE with significant selectivity toward BChE; compound 1b was 955-fold less potent for BChE than for AChE [21]. Cycloaddition was also used for the design and synthesis of a novel class of reactivators of AChE inhibited by nerve agents called oximes, bearing an AChE peripheral site anchor positioning group and a choline site binding group [12,20].

In a previous study, we tested the effect of triazole fungicide tebuconazole in the form of a commercially available formulation (Orius^®^ and Prosaro^®^) on the activity of cattle cholinesterase and observed a decrease in AChE and BChE activity in a dose-dependent manner [22]. In this work, we tested the type and potency of the anticholinesterase activity of the triazole mefentrifluconazole (Ravystar^®^) and carbamate pyraclostrobin, a component of the binary fungicidal mixture that also includes mefentrifluconazole (Ravycare^®^). A molecular docking study between mefentrifluconazole, pyraclostrobin and other mefentrifluconazole-related fungicides revealed residues of the lipophilic active site of AChE, and BChE indicated key interactions for ligand binding. Additionally, the physicochemical properties of the studied fungicides were compared to values for CNS-active drugs to estimate the blood–brain barrier (BBB) permeability together with oral bioavailability to evaluate the risk of brain anticholinesterase activity.

## 2. Results and Discussion

### 2.1. Inhibition of Cholinesterases by Fungicides

By determining the kinetic constants of inhibition for fungicides mefentrifluconazole and pyraclostrobin, the impact on cholinergic enzymes AChE and BChE was evaluated. Enzyme activity was measured at a range of inhibitor concentrations to determine the enzyme–inhibitor equilibrium dissociation constant (*K*_i_) or the second-order rate constant of inhibition (*k*_i_). The kinetic constants for the inhibition of human AChE and BChE with the tested fungicides are listed in Table 1.

**Figure 2:**
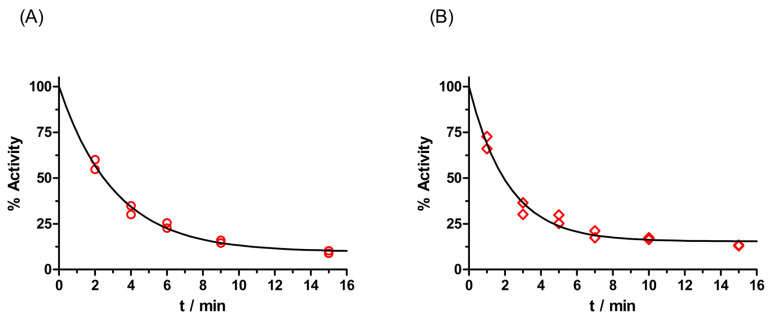
Representative plot of progressive inhibition of AChE (**A**) and BChE (**B**) by carbamate pyraclostrobin (c = 50 μM). The theoretical curve (black line) was calculated using Equation (2).

Both mefentrifluconazole and pyraclostrobin inhibited AChE and BChE, but differently. Mefentrifluconazole acted as a reversible inhibitor, while pyraclostrobin, a carbamate, progressively inhibited AChE and BChE [23]. No spontaneous reactivation of ChE was observed during 1 h of pyraclostrobin inhibition. Mefentrifluconazole was the more potent inhibitor of human AChE than BChE, which showed an about seven-fold lower affinity for mefentrifluconazole than AChE. Pyraclostrobin acted as a somewhat faster inhibitor of human BChE than AChE, ~40%, resulting in more than 90% of control activity inhibition within 15 min for both AChE and BChE (Figure 2).

In a previous study, the impact of triazole fungicide tebuconazole on cattle blood cholinesterase was determined. Tebuconazole in the form of a commercial fungicide formulation inhibited cattle AChE and BChE in a concentration-dependent manner at 144 μg mL^−1^ (*K*_i_ = 468 μM) for AChE and 135 μg mL^−1^ (*K*_i_ = 439 μM) for BChE [22]. Since AChE’s primary role is the regulation of cholinergic neurotransmission, its structure and amino acid composition of the hydrolytic active site are very conserved among various vertebrate species [24,25]. It is reasonable to expect that tebuconazole may also show an effect of inhibition of human AChE and BChE. Tebuconazole is structurally related to mefentrifluconazole where, instead of one halogenated benzene ring, two benzene rings are present in the form of phenoxy phenyl baring halogen atoms (see Figure 1).

The inhibition potential of mefentrifluconazole toward human AChE is comparable to that of herbicides bensulide, piperophos and desmedipham and about two-fold lower than the inhibition potential of phenmedipham and flufenacet [26]. Mefentrifluconazole showed a much smaller inhibition potential toward human BChE than 11 herbicides, up to 100-fold [26]. In a recent study on the progressive inhibition of human AChE and BChE by organophosphorus pesticides phosalone, ethoprophos, methamidophos and fenamiphos, it was shown that the bimolecular rate constant of inhibition with ethoprophos was ~30-fold higher than that of phosalone and methamidophos, and about 16-fold above the rate for fenamiphos [27]. The most potent inhibitor of BChE was fenamiphos, while ethoprophos followed as the second fastest inhibitor. The bimolecular rate constant of AChE inhibition with pyraclostrobin was above the ones for organophosphorus pesticides and about 10-fold lesser than the rate for ethoprophos (*k*_i_ ≈ 65,000 M^−1^ min^−1^), whereas in the case of BChE inhibition, it was above the rates for phosalone and methamidophos, and below those for ethoprophos (*k*_i_ ≈ 16,000 M^−1^ min^−1^) and fenamiphos (*k*_i_ ≈ 36,000 M^−1^ min^−1^) [27]. Additionally, the pyraclostrobin rate constant is about two-fold lower than the organophosphorus herbicide butamifos constant (*k*_i_ ≈ 17,000 M^−1^ min^−1^) [26].

### 2.2. Molecular Modeling of Fungicide Complexes with AChE and BChE

**Figure 3:**
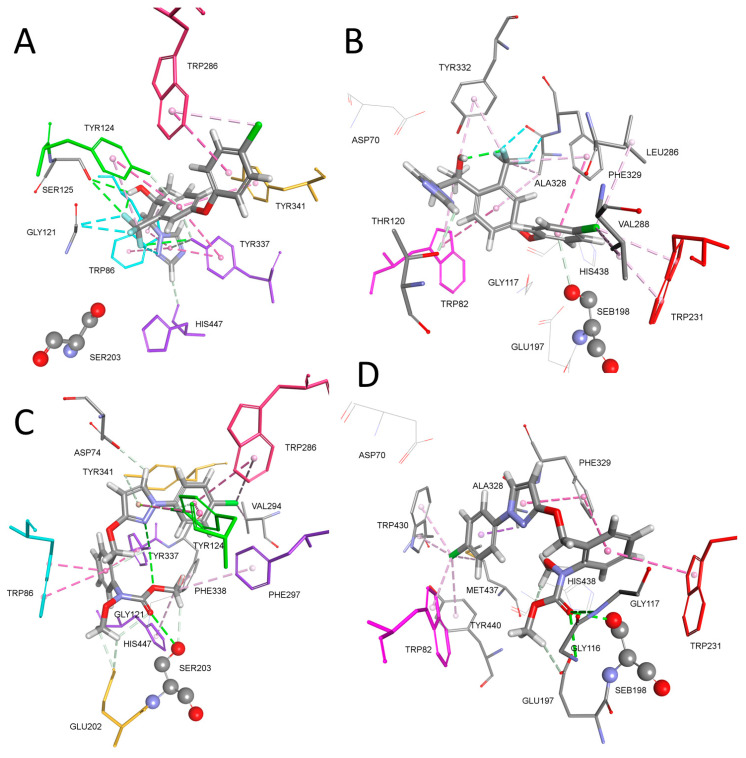
Molecular modeling of a complex between mefentrifluconazole and AChE (**A**) and BChE (**B**). Pyraclostrobin in complex with AChE (**C**) and BChE (**D**). The crystal structure of AChE was PDB code 4PQE [29] and of human BChE 2PM8 [30]. Fungicide interactions are represented as dashed lines: hydrophobic (purple), hydrogen bonds (green) and halogen (light blue).

The molecular modeling of a complex between AChE and mefentrifluconazole or pyraclostrobin, as well as a BChE complex with these fungicides, enabled a visualization of the interactions between the pesticide’s molecule and amino acids lining the enzyme active site gorge (Figure 3). Mefentrifluconazole was stabilized in the AChE active site by the interactions formed in the choline binding site (Trp86 and Tyr337) with a triazole ring and in the peripheral binding site (Tyr124 and Trp286) with a 4-chlorophenoxy phenyl group. The trifluoromethyl group forms halogen interactions with Gly121 from the oxyanion hole and additional hydrogen bonds with Ser125 and Tyr337. The complex between BChE and mefentrifluconazole revealed a binding pose where the 4-chlorophenoxy phenyl group was directed toward Trp231 in the acyl binding pocket, forming several hydrophobic interactions with Trp231, Leu286 and Val288. Additional interactions of the 4-chlorophenoxy phenyl group were with Ser198, Phe329, Ala328 and Trp82 from the choline binding site and with Tyr332 from the peripheral binding site. The trifluoromethyl group formed halogen interactions with Ala328 and hydrophobic interactions with Phe329 and Tyr332. The triazole ring was stabilized by a hydrogen bond with Thr120.

The model of the complex between AChE and the carbamate pyraclostrobin shows the orientation of pyraclostrobin in which the leaving group of the fungicide is positioned opposite to the oxygen (Oγ) of catalytic Ser203 and therefore creates the hydrogen bond with Ser203 (Figure 3C,D). This orientation is in accordance with a nucleophilic attack by the catalytic serine during enzyme progressive inhibition [26,27,28]. The methoxy group, which is a leaving group, is directed toward Phe295 from the acyl binding pocket, creating hydrophobic interactions with Phe295 and His438, and yielding a hydrogen bond with Ser203. The second methoxy group forms interactions with Glu202 and His438, while the benzene ring is stabilized in a π-π sandwich with residues of the choline binding site Trp86 and Tyr337. The pyrazole ring is stabilized in the middle of the AChE active site gorge forming a hydrogen bond with Asp74 from the peripheral binding site, Tyr337 and Tyr341. The stabilization of the 4-chlorophenyl group came from multiple hydrophobic interactions with Tyr124 and Trp286 from the peripheral binding site, as well as Val294.

The orientation of pyraclostrobin in the active site of BChE was also opposite to the oxygen (Oγ) of catalytic Ser198, where the oxygen from the carbonyl group created hydrogen bonds with Ser198 and with Gly116 and Gly117 from the oxyanion hole. The methoxy group created a hydrogen bond with Glu197. The benzene ring was stabilized via hydrophobic interactions from Trp231, a residue from the acyl binding pocket, and Phe329. Phe329 also stabilized the pyrazole ring, while Trp82, Ala328, Phe430, Met438 and Tyr440 stabilized the 4-chlorophenyl group via multiple hydrophobic interactions.

**Figure 4:**
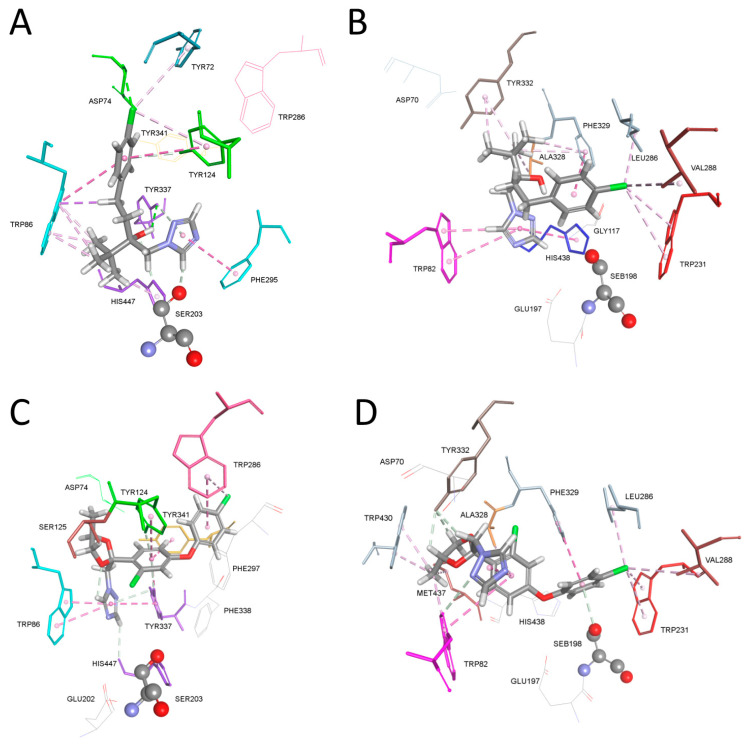
Molecular modeling of a complex between tebuconazole and AChE (**A**) and BChE (**B**). Difenoconazole in complex with AChE (**C**) and BChE (**D**). Crystal structure of AChE was PDB code 4PQE [29] and of human BChE was PDB code 2PM8 [30]. Fungicide interactions are represented as dashed lines: hydrophobic (purple) and hydrogen bonds (light green).

A comparison of the possible binding modes in the enzyme active site and potency of anticholinesterase activity, modeling of a complex between AChE or BChE with triazoles tebuconazole and difenoconazole, structurally related to mefentrifluconazole, was also performed (Figure 4). The binding pose of tebuconazole in the AChE active site showed a triazole position in the acyl pocket creating interaction with Phe297 and hydrogen bonds with Ser203 and Tyr337. The *tert*-butyl group was positioned close to Trp86 from the choline binding site, forming multiple hydrophobic interactions with Trp86 and His447. This pose resembled the binding of the choline part of AChE substrate acetyl thiocholine (PDB code 2C4H) [31]. The 4-chlorophenyl moiety created interactions with residues Tyr72, Asp74 and Tyr124 from the peripheral binding site, as well as with Trp86. Stabilization of tebuconazole in the BChE active site showed a π-π sandwich between the triazole ring, the indole ring of Trp82 and the imidazole ring of His447. In the acyl pocket, the 4-chloprophenyl group formed multiple hydrophobic interactions with Trp231, Leu286 and Val288 along with Phe329. Phe329 also stabilized the *tert*-butyl group together with Ala328 and Tyr332 from the peripheral site.

Difenoconazole is similar to mefentrifluconazole inasmuch that instead of a chlorine atom, mefentrifluconazole has a trifluoromethyl group, and difenoconazole’s oxygen atoms are included in its 1,3-dioxolan ring, while mefentrifluconazole has a free hydroxy group. The binding pose of difenoconazole in the AChE active site showed a π-π sandwich between the triazole ring, the indole ring of Trp86 and the 4-hydroxyphenyl ring of Tyr337. The triazole ring created a hydrogen bond with His447. The 4-chloprophenyl group formed multiple interactions with Tyr337 and Tyr341, as well as Tyr124 and Trp286 from the peripheral site. Difenoconazole in the BChE active site formed multiple hydrophobic interactions with Trp231, Leu286 and Val288 with its 4-chlorophenoxy phenyl group, and formed additional interactions with Ser198, Ala328 and Phe329. The bent conformation of difenoconazole enabled a π-π interaction between the triazole ring and the second benzene ring from the 4-chlorophenoxy phenyl group, as well as the stabilization of those rings in the choline binding site via hydrophobic interactions from Trp82. The 1,3-dioxolan ring bearing a methyl group was stabilized via multiple hydrogen bonds from Tyr332 and hydrophobic interactions from Trp82, Trp430 and Met437.

Interactions of other cholinesterase complexes with triazoles structurally related to mefentrifluconazole formed in the active site of AChE and BChE are listed in Table 2. Several modes of AChE and BChE binding are shown in Figures S2 and S3. In general, binding can be described based on the triazole fungicide groups’ location in the active site subdomains including the choline binding site, the acyl pocket and the peripheral binding site. In this set of triazoles, there was no interaction with Trp286 from the AChE peripheral binding site, meaning that the ligand was positioned in the lower part of the active site close to the choline binding site and the acyl pocket [28]. In case of epoxiconazole and fluconazole, which have a mono- or bi-fluorinated benzene ring, the fluorine atom was directed toward Tyr133 next to Trp86, creating a halogen interaction with Glu202. The triazole ring was stabilized in the middle of the active site gorge.

The binding pose of metconazole and propiconazole in the AChE active site showed a π-π sandwich between the triazole ring, the indole ring of Trp86 and the 4-hydroxyphenyl ring of Tyr337 as was determined for mefentrifluconazole and difenoconazole. The 4-chloropheny group of metconazole was stabilized by Tyr124, Tyr337 and Tyr341 in the middle of the active site gorge, while the propiconazole 2,4-dichlorophenyl group was directed toward the acyl pocket and stabilized by Phe295 together with Tyr124, Tyr337, Phe338 and Tyr341. Voriconazole is known for the treatment and prevention of fungal infection in humans, and its binding pose shows stabilization of the 5-fluoropyrimidine group in the choline binding site; moreover, the triazole ring forms a hydrogen bond with Ser203 and a hydrophobic interaction with Tyr337. The 2,4-difluorophenyl group is stabilized in the middle of the gorge, creating interactions with Asp74, Thr83, Tyr124 and Ser125.

Summarized interactions of triazoles in the BChE active site show the positioning of the triazole ring in the choline binding site and an alternative positioning in the acyl pocket in the case of metconazole and the second triazole ring of fluconazole toward Trp231. Only the binding pose of propiconazole did not show an interaction with catalytic Ser198 and glycine(s) from the oxyanion hole. All triazoles created a hydrophobic and/or a hydrogen bond with His438. Chlorinated benzene rings of epoxiconazole and propiconazole are stabilized in the acyl pocket via hydrophobic interaction with Trp231. The fluorinated benzene rings of epoxiconazole, fluconazole and voriconazole were directed toward Trp82 or the neighboring Asn83. Only fluconazole did not create a hydrophobic interaction with Phe329.

These types of binding interactions for triazole fungicides were known from the crystal structures of AChE or BChE and cholinesterase inhibitors (Figures S4 and S5). Sharpless et al. prepared the dual binding high-affinity AChE inhibitor *anti*-TZ2PA6 bearing the choline binding site moiety and the peripheral binding site moiety linked via a triazole ring. Mouse AChE-TZ2PA6 complex (PDB code 1Q84) showed a tacrine moiety stabilized via a π-π sandwich with Trp86 and Tyr337. The triazole ring was stabilized in the middle of the active site gorge by Tyr341, Tyr337 and Tyr124 and the propidium peripheral binding moiety was stabilized with Trp286, Tyr72 and Ser293 via multiple hydrophobic interactions (Figure S4A) [32]. AChE in complex with chlorinated ligand huprine x (PDB code 1E66) was stabilized at the choline binding site by Trp84 and Phe330 via a π-π sandwich, and the chlorine atom was stabilized by Trp432, Met436 and Ile439 including Phe330 (*Torpedo californica* AChE amino acid numbering) (Figure S4B) [33]. In the complex of AChE with the dichlorophenoxy phenyl piperidine ligand (PDB code 5FOQ), chlorine atoms are stabilized at the peripheral binding site with Phe286 (and Asp74), while the piperidine ring is stabilized with Trp86 and forms hydrophobic interactions with ligand benzene group next to His447 and in the middle of the gorge with Tyr337 and Tyr341 (Figure S4C) [34].

BChE in complex with the triazole–imidazole oxime ligand (PDB code 6T9P) stabilizes the imidazole ring with Trp82 via hydrophobic interactions and the neighboring triazole ring is stabilized by Tyr332 (Figure S5A) [12]. The crystal structure of the 2-PAM analogue with a pentafluoro benzyl group (PDB code 4B0P) showed a stabilization of the pyridinium ring by Trp82 and a stabilization of the pentafluoro benzyl group by Tyr332 from the peripheral binding site, as well as the formation of halogen interactions with Asp70, Pro285 and Ala328 (Figure S5B) [35]. BChE in complex with a chlorotacrine-tryptophan dual binding ligand (PDB code 6I0C) showed a stabilization of the chlorine atom near the choline binding site by Trp430 and Met437 and a stabilization of tacrine moiety between Trp82, Ala328 and His438 (Figure S5C) [36]. The tryptophan group was located at the acyl binding pocket, and Tyr332 stabilized the alkane linker via hydrophobic interactions. Another example was the complex with a hybrid triazole–huprine ligand (PDB code 7AIY), where the chlorine atom is stabilized at the acyl pocket and directed toward Trp231, Leu286 and Phe398. At the choline binding site, Trp82 stabilized the aliphatic part of the ligand, and the triazole ring was stabilized by Phe329 (Figure S5D) [37].

### 2.3. Triazoles as Potential Cholinesterase Inhibitors

Apart from the molecular modeling of complexes between triazoles and cholinesterases, a scoring function with the potential for predicting ligand potency to inhibit AChE was applied to evaluate the inhibition potency of triazoles in comparison to known anticholinesterases. CHARMm-based scoring function Cdocker interaction energy [38] was analyzed in a recent study on BChE ligands, whose BChE–ligand crystal structure was solved and enzyme–ligand interactions well defined. The scores derived from the scoring functions for each ligand were correlated to its inhibition constant. The average score value for set of 20 BChE ligands was 59 ± 12 kcal/mol [39].

**Figure 5:**
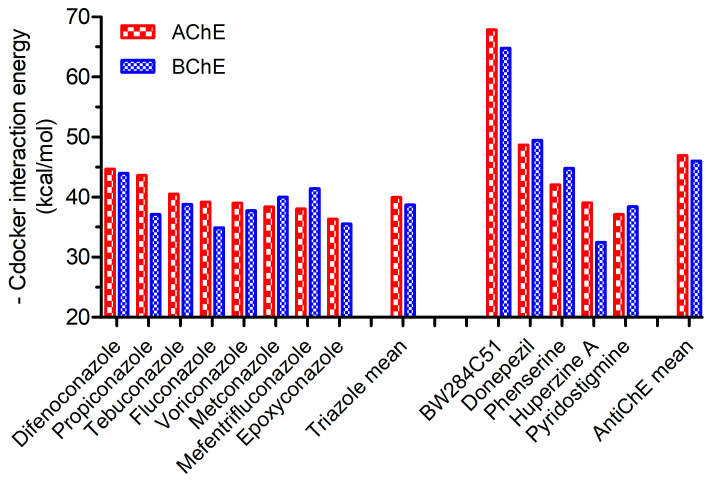
A comparison of interaction energies obtained for triazole fungicides and anticholinesterases after molecular docking into the active site of AChE (PDB code 4PQE [29]) and BChE (PDB code 2PM8 [30]). The energy of the top pose is listed after ranking by CHARMm-based scoring function CDOCKER Interaction Energy [38].

Here, scores of triazoles calculated by Cdocker interaction energy scoring function were obtained for both human AChE and BChE and compared to the scores obtained for anticholinesterases (Figure 5). Score values for triazoles ranged from 36.3 kcal/mol for epoxiconazole to 44.7 kcal/mol for difenoconazole for AChE and from 34.9 kcal/mol for fluconazole to 44.0 kcal/mol for difenoconazole for BChE. Score values for anticholinesterases ranged from 37.1 kcal/mol for pyridostigmine to 67.9 kcal/mol for BW284C51 for AChE and from 32.5 kcal/mol for Huperzine A to 64.8 kcal/mol for BW284C51 for BChE. A comparison of average values for two sets of compounds for AChE and BChE show slightly lower values obtained for triazoles, 39.97 ± 2.86 kcal/mol and 38.68 ± 3.05 kcal/mol, than for anticholinesterases, 46.95 ± 12.49 kcal/mol and 46.0 ± 12.31 kcal/mol, for AChE and BChE, respectively. This is expected since triazoles are not designed to be inhibitors of cholinesterases. Nevertheless, the scoring values for triazole fungicides indicated the potency of the studied triazoles to show anticholinesterase activity.

### 2.4. In Silico Evaluation of CNS Activity

A promising drug candidate should have sufficient efficacy against the therapeutic target and show appropriate ADME (absorption, distribution, metabolism and excretion) properties at a therapeutic dose. Therefore, ADME including toxicity plays a key role in drug discovery and development. Voriconazole and clotrimazole are drugs characterized by good adsorption properties, and this is also characteristic for other triazole fungicides due to application in plant protection management [3]. In the drug design of CNS-active drugs, beneficial properties are oral adsorption and the ability of the compounds to cross the BBB leading to CNS activity [40,41]. By studying numerous approved drugs, Pajouhesh and Lenz proposed general rules for medicinal chemical properties of successful CNS drugs, including physicochemical properties together with ADME properties [42].

**Figure 6:**
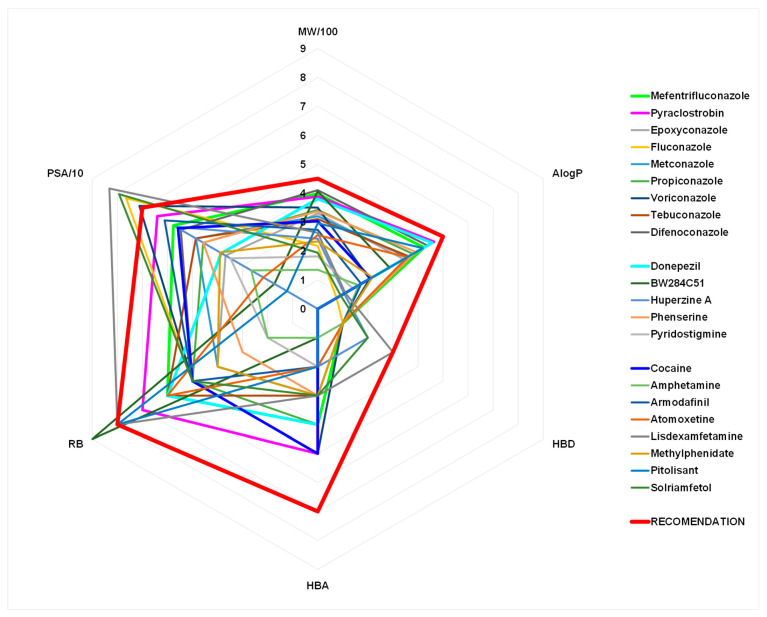
Radar plot of physicochemical properties (molecular weight, MW; lipophilicity coefficient, logP; number of hydrogen bonds donors, HBD, and acceptors HBA; rotatable bonds, RB; polar surface area, PSA) of the studied compounds (Table S1, Figure S1). Recommended values for CNS-active drugs are presented by a red line [42].

**Figure 7:**
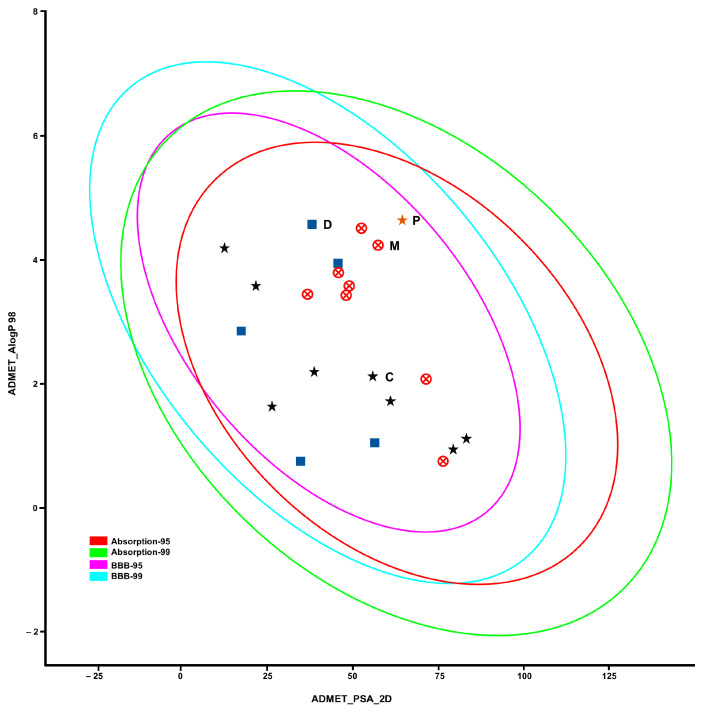
Blood–brain barrier permeability plot. ADME (absorption, distribution, metabolism and excretion) properties of the studied compounds were obtained; calculated lipophilicity (AlogP) and polar surface area (PSA) are correlated (Table S1). Mefentrifluconazole (M) and other triazoles are represented as red crossed circles, pyraclostrobin (P) is gold star, donepezil (D) and other anticholinesterases are blue squares and cocaine (C) and other CNS-active compounds are black stars. The areas within magenta (BBB-95) and red (Absorption-95) ellipses represent compounds with good BBB permeability and human intestinal absorption [41].

To evaluate the ability of triazole fungicides to cross the BBB to achieve their anticholinesterase activity on AChE and BChE in the brain, the six physicochemical properties were calculated and compared to physicochemical properties for known anticholinesterase [11] and CNS-active compounds [40,41] (Figure 6 and Figure S1). The plot of lipophilicity (AlogP) and polar surface area (PSA) estimated BBB penetration ability of triazoles in comparison to anticholinesterase and CNS-active drugs (Figure 7). In silico prediction tests were performed on triazoles to see whether these compounds possess high bioavailability for sufficient penetration through biological barriers. The prediction showed that the compounds are suitable for oral ingestion, causing increasing concentration and residency in the brain.

## 3. General Discussion

We showed that mefentrifluconazole and pyraclostrobin are potent inhibitors of cholinesterases which differ in the type of ChE inhibition. Mefentrifluconazole showed higher inhibition potency of human AChE compared to BChE, while pyraclostrobin was a ~40% faster inhibitor of human BChE than AChE. Molecular modeling of cholinesterase complexes with mefentrifluconazole and triazoles structurally related to it bind to the AChE/BChE active site through simultaneous interactions with amino acids from PAS and CAS as dual binding site inhibitors. In silico analysis using CHARMm-based scoring function Cdocker interaction energy showed that triazoles structurally related to mefentrifluconazole may have moderate anticholinesterase activity after comparison to known anticholinesterase compounds. Moreover, in silico evaluation of CNS activity showed that all triazoles may also have the ability to cross the BBB as the main requirement for potentially CNS-active compounds.

**Figure 8:**
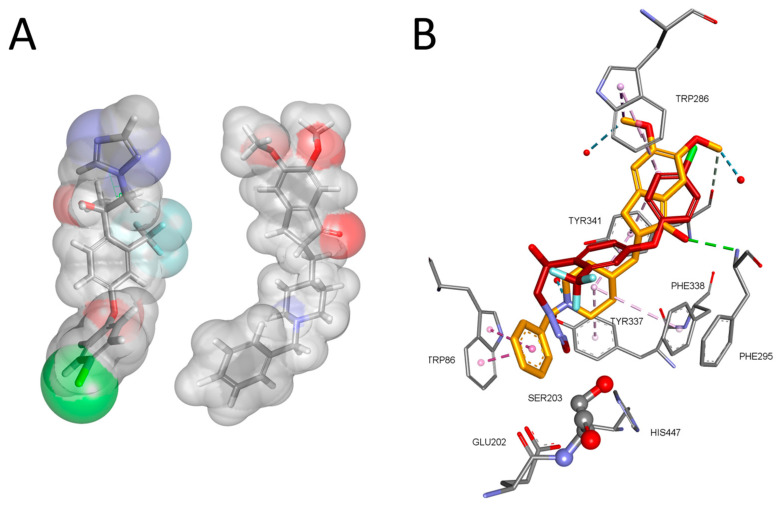
Comparison of 3D structures of mefentrifluconazole (**left**) and AChE inhibitor donepezil (**right**) (**A**). Comparison of binding pose in human AChE of donepezil (carbon atoms are gold; PDB code 6O4W) [43] and mefentrifluconazole (carbon atoms are dark red; see Figure 3A) (**B**).

Comparison of molecular conformation of mefentrifluconazole and the known anti-Alzheimer drug donepezil show similarity in size, three-ring composition and hetero atom presence (Figure 8). This means that molecular properties can be linked to anticholinesterase activity as shown by triazole molecular docking.

In a previous study, a QSAR regression model for estimating the potency of compounds to inhibit AChE was developed [44]. The predictive power of the model with only three simple descriptors was tested and confirmed on 165 compounds in total. Moreover, the QSAR regression model for BChE developed on a set of 297 compounds also with three simple descriptors, different from those for the AChE model, showed the same level of predictive power [39]. The approach of multi-target drug development (MTDD) is based on the idea that compounds interacting with more than one biological target cause the balance of wanted activities to maximize efficacy and safety. The optimal activity ratio is important for a designed multiple ligand to interact specifically with multiple targets [45,46]. A QSAR regression model in combination with MTDD can be used for ligand design with improved efficacy, but it can also be used to avoid molecular fragments in the lead design, causing activity toward unwanted biological targets. This means that in the development of future biologically active compounds, in silico methods can be used to test the possible diversity of biological activity in response to unwanted biological targets that are already characterized by drug design and ligand activity/inhibition regression models.

Another important feature of biologically active compounds is the species selectivity, such as the selectivity of mefentrifluconazole toward fungi enzyme in comparison to human CYP stated here. Knowledge about the structure and function of biological targets from various species can be used in combination with in silico methods to increase species activity and selectivity. For example, inhibition of human, mouse, and horse BChEs by the carbamate bambuterol showed 27-fold higher inhibition rate for mouse BChE than horse BChE [47]. Development of species-selective active compounds will minimize negative effects or threats to other species including humans, insects or aquatic species affected by plant disease management chemicals.

## 4. Materials and Methods

### 4.1. Chemicals

Mefentrifluconazole (2-[4-(4-chlorophenoxy)-2-(trifluoromethyl)phenyl]-1-(1,2,4-triazol-1-yl)propan-2-ol) was purchased from AmBeed (Arlington Heights, IL, USA), pyraclostrobin (methyl N-[2-[[1-(4-chlorophenyl)pyrazol-3-yl]oxymethyl]phenyl]-N-methoxycarbamate) was purchased from Tebubio (Le Perray-en-Yvelines, France) and methanol was obtained from Merck (Darmstadt, Germany). Stock solutions of mefentrifluconazole (100 mM) and pyraclostrobin (10 mM) were prepared in methanol and stored at 4 °C. Further dilutions were also prepared in methanol for reversible and progressive inhibition. Acetylthiocholine iodide (ATCh), and thiol reagent 5,5′-dithiobis(2-nitrobenzoic acid) (DTNB) were purchased from Sigma-Aldrich (St. Louis, MO, USA). A stock solution of ATCh (20 mM) was prepared in water and DTNB (6 mM) was prepared in sodium phosphate buffer (0.1 M, pH 7.4). All chemicals were of analytical grade with a declaration of purity above 98%.

### 4.2. Enzymes

Recombinant human AChE and human BChE were a gift from Dr X. Brazzolotto and Dr F. Nachon (D’epartement de Toxicologie et Risques Chimiques, Institut de Recherche Biom’edicale des Arm’ees, Bretigny-sur-Orge, France). Enzymes were stored at 4 °C.

### 4.3. Inhibition Measurements

The activity of AChE and BChE was assayed by the Ellman spectrophotometric method [48,49], using ATCh-iodide as a substrate (concentration range 0.1–0.8 mM for reversible inhibition, 0.5 mM for progressive inhibition) and the thiol reagent DTNB (0.3 mM, ε = 14,250 dm^3^ mol^−1^ cm^−1^; [50]) in the presence of mefentrifluconazole (concentration range 50–500 μM) and pyraclostrobin (concentration range 10–100 μM). For progressive inhibition, the increase in the absorbance of the TNB anion was measured in a 0.1 M sodium phosphate buffer (pH 7.4) at 25 °C and 412 nm in 3 mL total volume quartz cuvettes on a CARY 300 spectrophotometer (Varian Inc., Belmont, Australia) with a temperature controller or in 96-well plates on an Infinite M200PRO plate reader (Tecan Austria GmbH, Salzburg, Austria) for reversible inhibition. Absorption measurements lasted up to 2 min for progressive inhibition and up to 5 min for reversible inhibition. Compound-induced ATCh-iodide nonenzymatic hydrolysis was not observed.

Due to the poor solubility of mefentrifluconazole and pyraclostrobin in phosphate buffer, compounds were dissolved and further diluted in methanol. The final concentration of methanol in the reaction mixture for all measurements was kept constant at 6% *v/v* to improve compound solubility [26,27].

Enzyme activity for reversible inhibition with mefentrifluconazole was measured at four substrate concentrations for five compound concentrations in triplicate. The experimentally obtained initial reaction rates at different substrate concentrations (ATCh) were fitted to Michaelis–Menten kinetics, wherefrom *K*_m_ and *V*_m_ were obtained by nonlinear regression using GraphPad Prism6 software (GraphPad, San Diego, CA, USA).

The enzyme–inhibitor dissociation constant, *K*_i_, and related kinetic parameters were evaluated from the competitive model of inhibition (Equation (1)),

(1) $$v_{i}=\frac{V_{m}\cdot S}{K_{m}\left(1+\frac{i}{K_{i}}\right)+S}$$

where *v*_i_ stands for enzyme activity in the presence of inhibitor (*i*), *V*_m_ is maximal enzyme activity, *K*_m_ is the Michalis constant and *K*_i_ is the enzyme–inhibitor dissociation constant.

For progressive inhibition, AChE and BChE were incubated up to 1 h with pyraclostrobin (10–100 μM) in a mixture of buffer and DTNB. Enzyme activity was measured at designated times upon the addition of ATCh using the Ellman method [48,49]. The rate constant of enzyme inhibition, *k*_i_, was evaluated from the reaction half time (t_1/2_) derived from the one-phase exponential decay model (Equation (2)) by nonlinear regression using GraphPad Prism6 software (GraphPad, San Diego, CA, USA),

(2) $$v_{i}=v_{o}\cdot e^{-k_{i}\cdot i\cdot t}\;\;\;\;\;\;\;\;\;t_{1/2}=ln2/\left(k_{i}\cdot i\right)\;$$

where *v*_0_ and *v*_i_ stand for enzyme activity in the absence and the presence of inhibitor (i) at time t, when *k*_i_ was the second-order inhibition rate constant and t_1/2_ a half time of a reaction.

### 4.4. In Silico Evaluation of Blood–Brain Barrier Permeability

The 3D structures of compounds analyzed in this study were obtained from public database PubChem and were minimized with the MMFF94 force field using ChemBio3D Ultra 12.0 (PerkinElmer, Inc., Waltham, MA, USA) (Figure S1). Three groups of structures used were azoles, anticholinesterase drugs and CNS-active compounds. The Discovery Studio 21.1 (BioVia, San Diego, CA, USA) ADMET descriptor protocol was used for the calculation of the following molecular parameters: lipophilicity coefficient, AlogP98 and topological polar surface area, PSA 2D. The correlation of parameters AlogP98 and PSA 2D was used for the prediction of blood–brain barrier permeability based on a training set of known CNS-active compounds with good adsorption and blood–brain barrier permeability properties. Confidence ellipses were generated from the absorption model derived from over 800 compounds that enter the CNS after oral administration developed by Egan and Lauri [41]. In addition to parameter AlogP98 and PSA 2D, molecular weight, MW, hydrogen bond donor, HBD, and hydrogen bond acceptor, HBA, were calculated for triazoles, anticholinesterase and the CNS-active compounds using Discovery Studio 21.1 (BioVia, San Diego, CA, USA) Calculate molecular properties protocol (Table S1). The calculated parameters were compared to recommended values for CNS-active drugs [42].

### 4.5. Computational Molecular Docking

The Discovery Studio 21.1 (BioVia, San Diego, CA, USA) Dock Ligands protocol (CDOCKER) with a CHARMm force field was used for the fungicide docking study in the active site of human AChE and BChE [51,52]. Crystal structures of human AChE PDB code 4PQE [29] and BChE PDB code 2PM8 [30] were used. The binding site within the AChE or BChE crystal structure was defined as the largest cavity surrounded by a sphere with a 13 Å radius, and it was used as the rigid receptor [12,27]. The final fungicide poses were ranked by scores derived by scoring functions CDOCKER Energy and CDOCKER Interaction Energy. Details about ligand docking using the CDOCKER protocol and subsequent scoring of generated ligand poses by CHARMm energy were described previously [39,53].

## 5. Conclusions

Measurement of cholinesterase activity in the presence of mefentrifluconazole and pyraclostrobin showed their anticholinesterase activity. Commercially available formulations of fungicide mefentrifluconazole (Revysol^®^) and binary mixture with pyraclostrobin (Revycare^®^) possess high concentrations of the active ingredient (≥100 g/L) and therefore may represent a threat not only to the human cytochrome but also to AChE, by disrupting cholinergic neurotransmission, or to the related BChE. Here, using an in silico study, we determined key interactions formed between triazoles and active site residues of AChE and BChE. Additionally, the in silico study suggested the high potential of triazole fungicides to cross the BBB and have CNS activity in combination with good oral adsorption properties. Therefore, triazole fungicides may have a negative impact on the brain as well as on peripheral AChE and BChE. In addition, Cdocker interaction energy scoring values for triazole fungicides indicate the potency of studied triazoles to show anticholinesterase activity due to the overlap with scores of known cholinesterase inhibitors. Our results lead to the approach in which a combination of in silico activity/property evaluation and a kinetic study may help in the future development of biologically active compounds, where in silico methods can be used to test the possible unwanted biological activity toward non-primary biological targets, already characterized by drug design and QSAR regression models including ChEs.

## Figures and Tables

**Table 1:** Constants (±SEM) for inhibition of human acetylcholinesterase (AChE) and human butyrylcholinesterase (BChE) by the tested fungicides. The enzyme–inhibitor equilibrium dissociation constant (*K*_i_) and the second-order rate constant of inhibition (*k*_i_) were determined from at least three experiments at 25 °C. Methanol concentration was 6% *v*/*v* in the Ellman reaction mixture.

Fungicide	*K*_i_ (μM)	*k*_i_ (M^−1^ min^−1^)
	AChE	
Mefentrifluconazole	101 ± 19	/
Pyraclostrobin	/	6496 ± 822 *
	BChE	
Mefentrifluconazole	653 ± 158	/
Pyraclostrobin	/	9242 ± 1343 *

* The rate constant of inhibition was determined at 50 μM pyraclostrobin.

**Table 2:** Key interactions of azole fungicides and the carbamate pyraclostrobin in the human AChE (PDB code 4PQE [29]) and BChE (PDB code 2PM8 [30]) active site gorge evaluated by molecular docking.

Compound	AChE			BChE		
	H-Bond	π-Orbitals	Other	H-Bond	π-Orbitals	Other
Mefentrifluconazole	Tyr124, Ser125, Tyr337, His447	Trp86, Tyr124, Trp286, Tyr337, Tyr341	Gly121 (Halogen acceptor)	Thr120, Ser198	Trp82, Gly116, Pro285, Tyr332, His438	Asp70 (Electrostatic)
Difenoconazole	Tyr124, Ser125, Tyr337, His447	Trp86, Tyr124, Trp286, Tyr337, Tyr341	/	Trp82, Ser198, Tyr332	Trp82, Trp231, Phe329, Trp430	Leu286, Val288, Met437 (Alkyl)
Epoxyconazole	Tyr124, Ser125, Tyr133, Ser203, Tyr337	Trp86, Tyr337	Glu202 (Halogen acceptor)	Glu197, Ser198, His438	Trp82, Trp231, Ala328, Phe329	Gly115, Gly116 (Amide π-stacked)
Fluconazole	Asp74, Tyr124, Ser125, Tyr133, Ser203, Tyr337, Tyr341	Trp86, Tyr337	Glu202 (Halogen acceptor)	Gly115, Gly117, Thr120, Ser198, His438	Trp82, Trp231	Gly116 (Halogen acceptor), Glu197 (Electrostatic)
Metconazole	Tyr124, Tyr337, His447	Trp86, Tyr124, Tyr337, Tyr341, His447	/	Asn83, Gly116, Thr120, Ser198	Trp231, Phe329, His438	/
Propiconazole	Tyr124, Tyr337, His447	Trp86, Tyr124, Phe297, Tyr337, Phe338, Tyr341	Pro88 (Alkyl)	His438	Trp82, Trp231, Ala328, Phe329	Leu286 (Alkyl)
Tebuconazole	Asp74, Tyr124, Ser203, Tyr337, His447	Tyr72, Trp86, Tyr124, Phe295, His447	/	/	Trp82, Trp231, Phe329, Tyr332, His438	Leu286, Val288, Ala328 (Alkyl)
Voriconazole	Asn87, Tyr124, Ser125, Ser203, Tyr337, His447	Trp86, Tyr124, Tyr337	Tyr72, Asn87 (Halogen acceptor)	Asp70, Asn83, Glu197, Ser198, His438	Trp82, Phe329, Tyr332, His438	Gly115 (Halogen acceptor), Glu197 (Electrostatic)
Pyraclostrobin	Asp74, Tyr124, Glu202, Ser203, Tyr337, Tyr341	Trp86, Tyr124, Trp286, Val294, Phe297, Phe338, His447	/	Gly116, Gly117, Glu197, Ser198	Trp82, Trp231, Ala328, Phe329, Trp430, Tyr440	Met437 (Alkyl)

## Data Availability

Data are available on request.

## Supplementary Materials

The following supporting information can be downloaded at: https://www.mdpi.com/article/10.3390/ijms25126310/s1.
