# Supplementary material for: Evaluation of Anticholinesterase Activity of the Fungicides Mefentrifluconazole and Pyraclostrobin
Source: Int J Mol Sci. 2024 Jun 7;25(12):6310. doi: 10.3390/ijms25126310 (PMC11204243; doi:10.3390/ijms25126310)
Supplement: Supplementary file 1 [file ijms-25-06310-s001.zip › ijms-3002840-Supplementary material.pdf]

Supplementary material

Evaluation of anticholinesterase activity for the fungicides mefentrifluconazole and pyraclostrobin

Dora Kolić and Goran Šinko*

Institute for Medical Research and Occupational Health, Ksaverska cesta 2, HR-10 000 Zagreb, Croatia

*Corresponding author:

Goran Šinko, e-mail: gsinko@imi.hr

Contents

Figure S1. Molecular structure of triazole compounds, pyraclostrobin, anti-cholinesterase compounds and central nervous stimulants used in this study. 2

Table S1. Physicochemical parameters relevant for CNS activity, calculated for triazole fungicides, carbamate pyraclostrobin, anti-cholinesterase and CNS-active compounds. 3

Figure S2. Molecular modeling of complex between epoxiconazole and AChE (A), and BChE (B) 4

Figure S3. Molecular modeling of complex between propiconazole and AChE (A), and BChE (B) 5

Figure S4. Crystal structure of complex between AChE and anti TZ2PA6 PDB code 1Q84 (A) 6

Figure S5. Crystal structure of complex between BChE and triazole-imidazole oxime ligand PDB code 6T9P 7

References 8

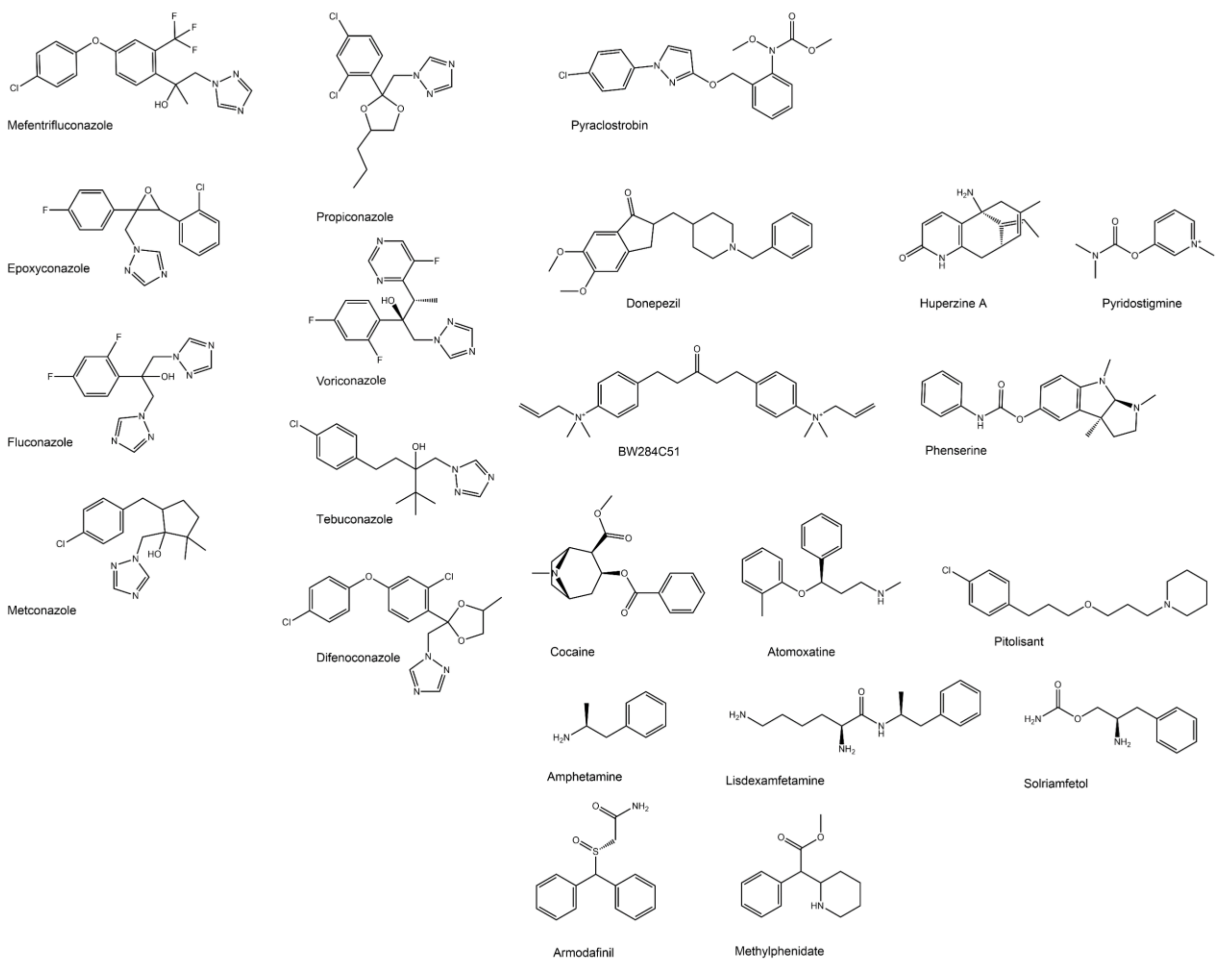

Figure S1. Molecular structure of triazole compounds, pyraclostrobin, anti-cholinesterase compounds and central nervous stimulants used in this study.

Table S1. Physicochemical parameters relevant for CNS activity, calculated for triazole fungicides, carbamate pyraclostrobin, anti-cholinesterase and CNS-active compounds. The recommendation values for molecular properties relevant for CNS activity are listed in the last row [S1].

Compound	Mol. weight (1/100)	AlogP	H-donor	H-acceptor	Rot. bonds	Polar sur. area (1/10)
Triazole						
Mefentrifluconazole	3.98	4.24	1	4	6	5.76
Pyraclostrobin	3.88	4.64	0	5	7	6.41
Epoxyconazole	3.3	3.43	0	3	4	3.68
Fluconazole	2.18	0.76	1	5	5	7.66 [1]
Metconazole	3.2	3.58	1	3	4	4.87
Propiconazole	3.42	3.8	0	4	5	4.57
Voriconazole	3.5	2.07	1	5	5	7.12 [1]
Tebuconazole	3.1	3.63	1	3	6	4.87
Difenoconazole	4.1	4.38	0	5	5	5.47
Anti-cholinesterase						
Donepezil	3.8	4.57	0	4	6	3.85
BW284C51	4.07	2.86	0	1	12 [1]	1.73
Huperzine A	2.42	1.06	2	2	0	5.67
Phenserine	3.37	3.93	1	3	3	4.57
Pyridostigmine	1.81	1.4	0	2	2	3.49
CNS-active						
Cocaine	3.03	2.12	0	5	5	5.58
Amphetamine	1.35	1.63	1	1	2	2.65
Armodafinil	2.73	1.73	1	2	5	6.11
Atomoxetine	2.55	3.58	1	2	6	2.17
Lisdexamfetamine	2.63	1.1	3	3	8	8.32 [1]
Methylphenidate	2.33	2.18	1	3	4	3.9
Pitolisant	2.96	4.19	0	2	8	1.23
Solriamfetol	1.94	0.92	2	3	5	7.93 [1]
Recommendation	4.5	5	3	7	8	7

[1] Values above recommendations.

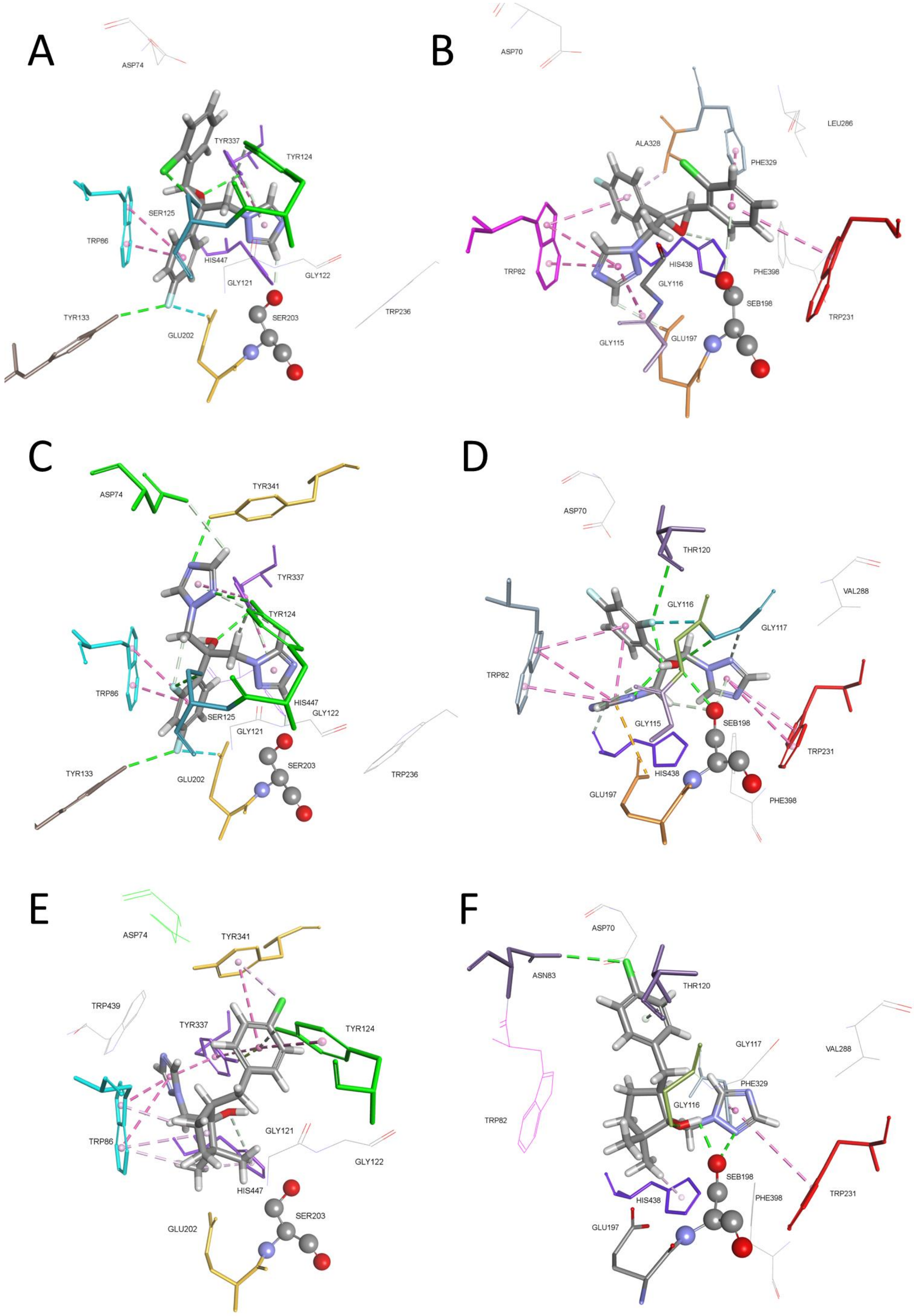

Figure S2. Molecular modeling of complex between epoxiconazole and AChE (A), and BChE (B), fluconazole in complex with AChE (C) and BChE (D), and metconazole in complex with AChE (E) and BChE (F). Crystal structure of human AChE was PDB code 4PQE [S2] and of human BChE was PDB code 2PM8 [S3]. Fungicide interactions are represented as dashed lines: hydrophobic (purple), hydrogen bonds (green) and halogen (light blue).

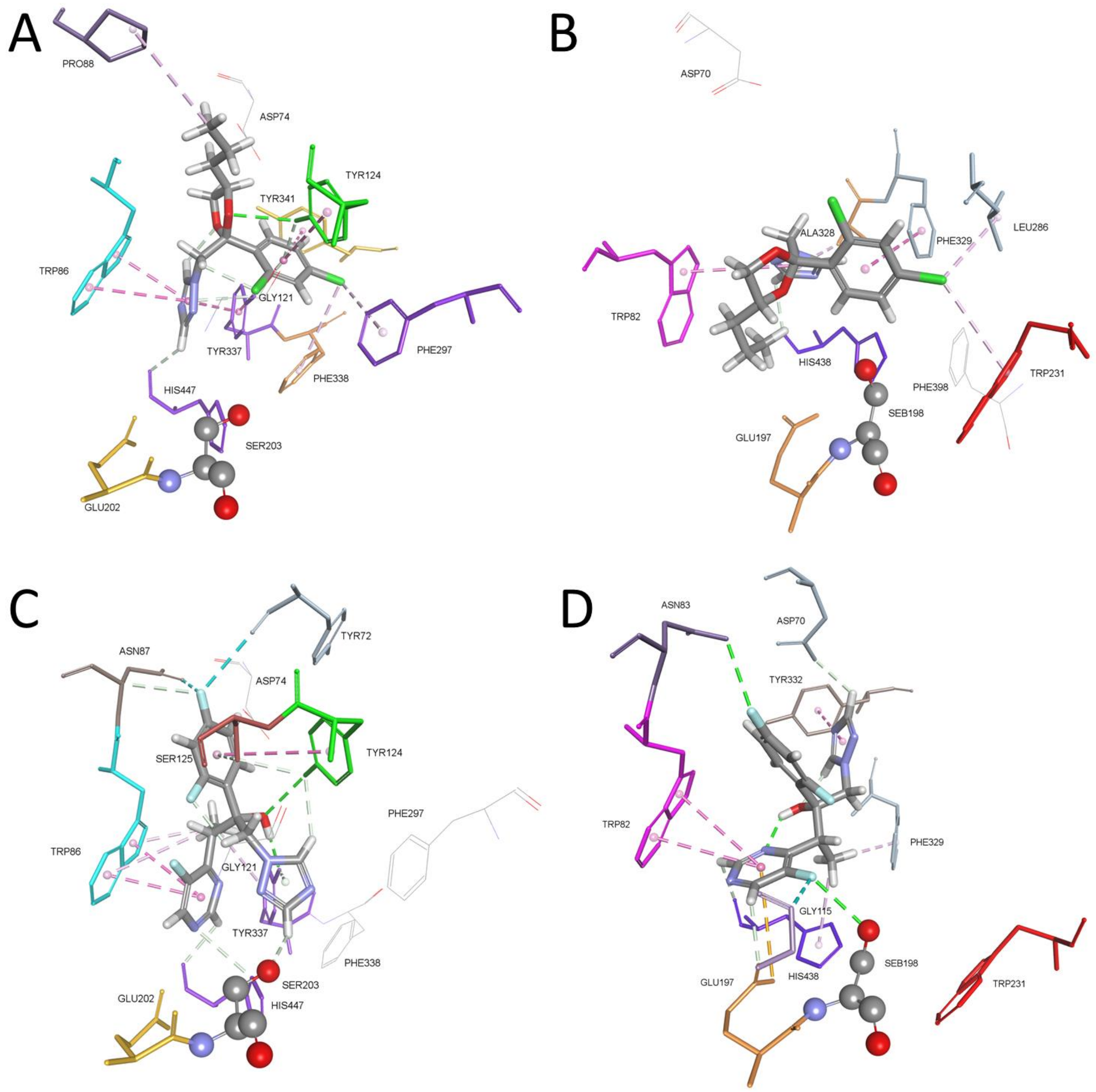

Figure S3. Molecular modeling of complex between propiconazole and AChE (A), and BChE (B), and voriconazole in complex with AChE (C) and BChE (D). Crystal structure of human AChE was PDB code 4PQE [S2] and of human BChE was PDB code 2PM8 [S3]. Fungicide interactions are represented as dashed lines: hydrophobic (purple), hydrogen bonds (green) and halogen (light blue).

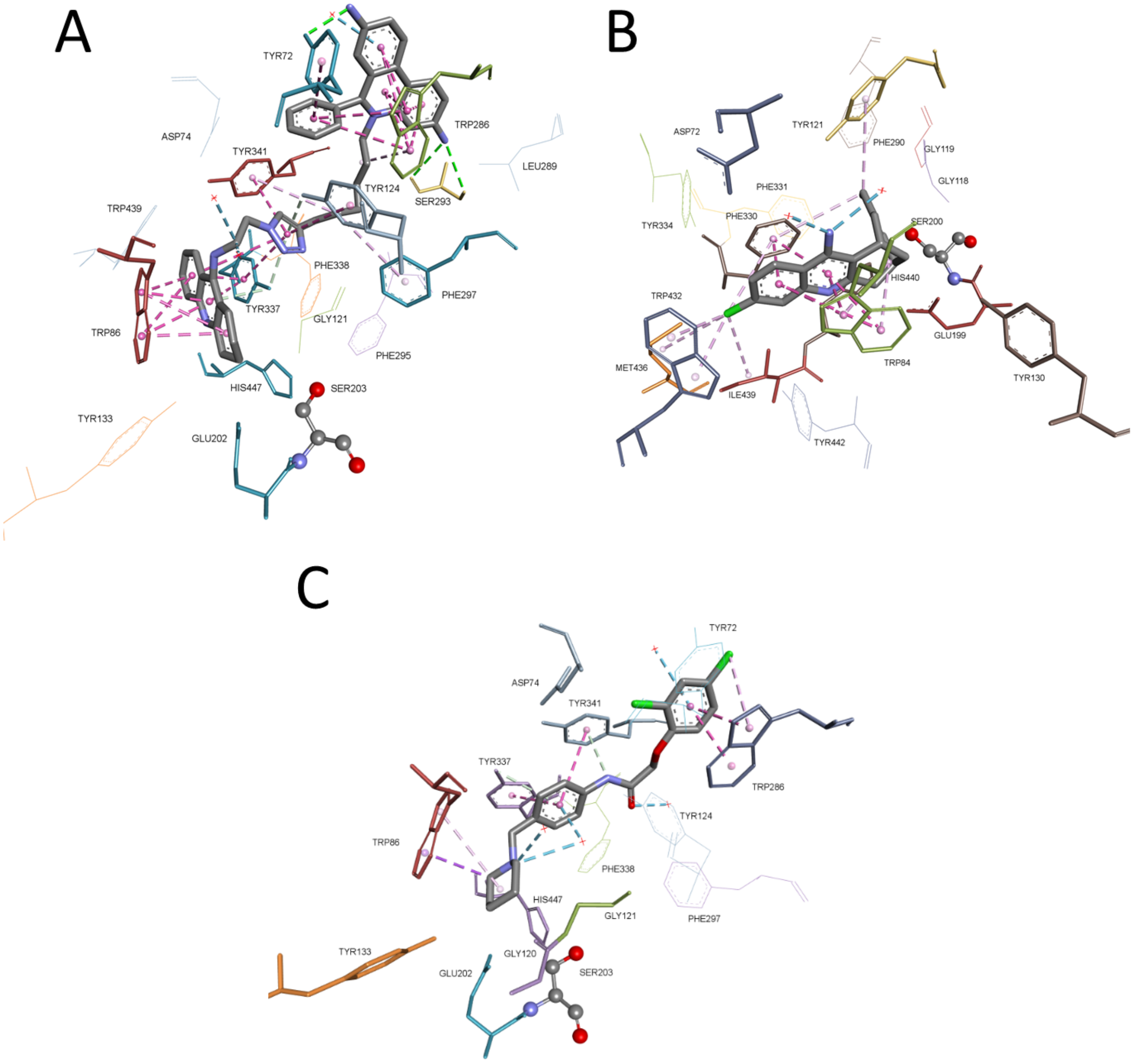

Figure S4. Crystal structure of complex between AChE and anti TZ2PA6 PDB code 1Q84 (A) [S4], huprine x PDB code 1E66 (B) [S5], dichlorophenoxy phenyl piperidine ligand PDB code 5FOQ (C) [S6]. Ligand interactions are represented as dashed lines: hydrophobic (purple), hydrogen bonds (green) and halogen (light blue).

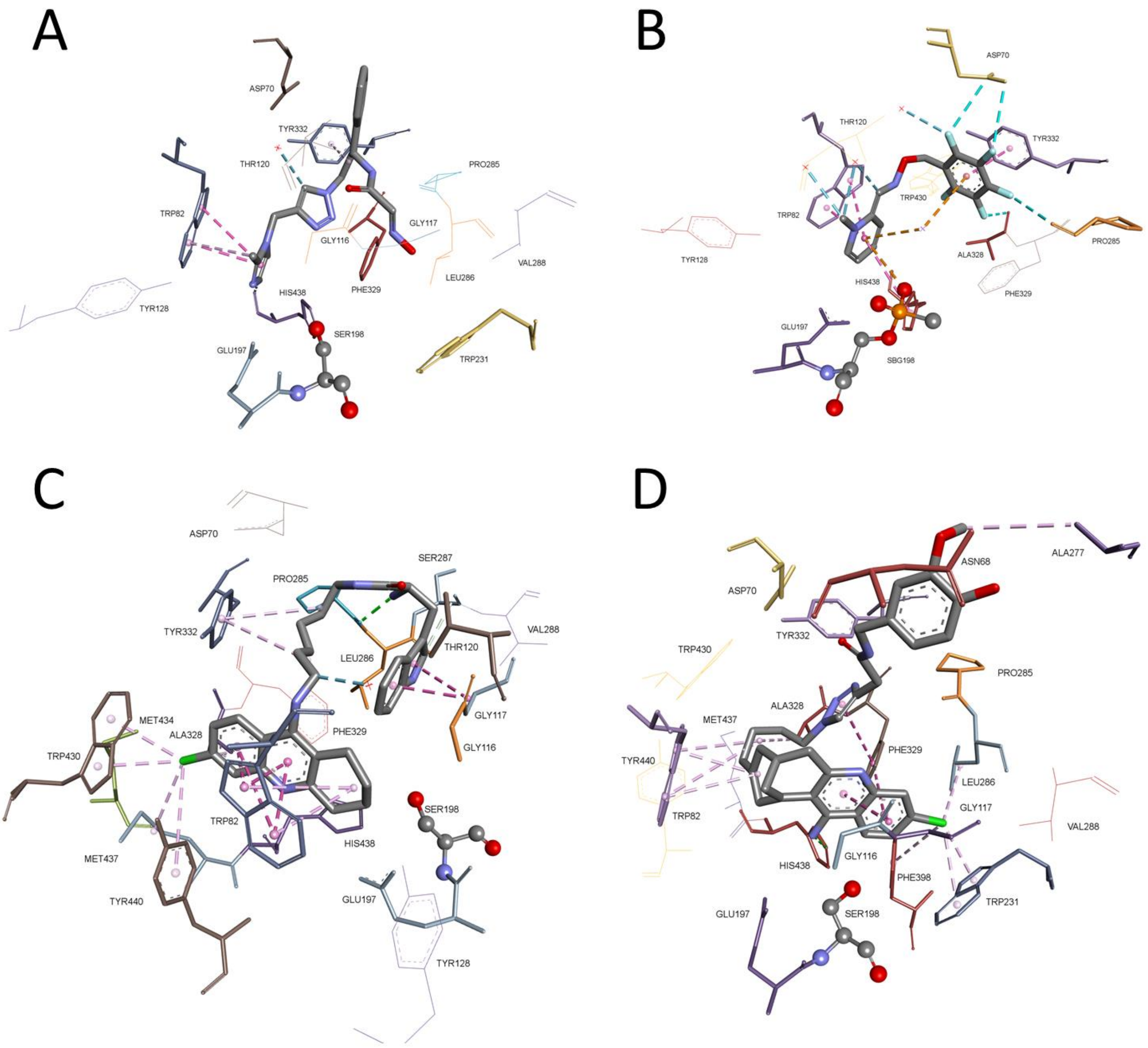

Figure S5. Crystal structure of complex between BChE and triazole-imidazole oxime ligand PDB code 6T9P (A) [S7], 2-PAM analogue with pentafluoro benzyl group PDB code 4B0P (B) [S8], chlorotacrine-tryptophan dual binding ligand PDB code 6I0C (C) [S9], hybrid triazole-huprine ligand PDB code 7AIY (D) [S10]. Ligand interactions are represented as dashed lines: hydrophobic (purple), hydrogen bonds (green) and halogen (light blue).

References

S1. Pajouhesh, H.; Lenz, G.R. Medicinal Chemical Properties of Successful Central Nervous System Drugs. *NeuroRX* **2005**, *2*, 541–553, doi:10.1602/neurorx.2.4.541.

S2. Dym, O.,; Unger, T.,; Toker, L.,; Silman, I.,; Sussman, J.L.,; Israel Structural Proteomics Center (ISPC) Crystal Structure of Human Acetylcholinesterase 2015.

S3. Ngamelue, M.N.; Homma, K.; Lockridge, O.; Asojo, O.A. Crystallization and X-Ray Structure of Full-Length Recombinant Human Butyrylcholinesterase. *Acta Crystallogr Sect F Struct Biol Cryst Commun* **2007**, *63*, 723–727, doi:10.1107/S1744309107037335.

S4. Bourne, Y.; Kolb, H.C.; Radic, Z.; Sharpless, K.B.; Taylor, P.; Marchot, P. Freeze-Frame Inhibitor Captures Acetylcholinesterase in a Unique Conformation. *Proceedings of the National Academy of Sciences* **2004**, *101*, 1449–1454, doi:10.1073/pnas.0308206100.

S5. Dvir, H.; Wong, D.M.; Harel, M.; Barril, X.; Orozco, M.; Luque, F.J.; Muñoz-Torrero, D.; Camps, P.; Rosenberry, T.L.; Silman, I.; et al. 3D Structure of *Torpedo Californica* Acetylcholinesterase Complexed with Huprine X at 2.1 Å Resolution: Kinetic and Molecular Dynamic Correlates ,. *Biochemistry* **2002**, *41*, 2970–2981, doi:10.1021/bi011652i.

S6. Berg, L.; Mishra, B.K.; Andersson, C.D.; Ekström, F.; Linusson, A. The Nature of Activated Non-classical Hydrogen Bonds: A Case Study on Acetylcholinesterase–Ligand Complexes. *Chemistry – A European Journal* **2016**, *22*, 2672–2681, doi:10.1002/chem.201503973.

S7. Maraković, N.; Knežević, A.; Rončević, I.; Brazzolotto, X.; Kovarik, Z.; Šinko, G. Enantioseparation, in Vitro Testing, and Structural Characterization of Triple-Binding Reactivators of Organophosphate-Inhibited Cholinesterases. *Biochemical Journal* **2020**, *477*, 2771–2790, doi:10.1042/BCJ20200192.

S8. Wandhammer, M.; de Koning, M.; van Grol, M.; Loiodice, M.; Saurel, L.; Noort, D.; Goeldner, M.; Nachon, F. A Step toward the Reactivation of Aged Cholinesterases – Crystal Structure of Ligands Binding to Aged Human Butyrylcholinesterase. *Chem Biol Interact* **2013**, *203*, 19–23, doi:10.1016/j.cbi.2012.08.005.

S9. Chalupova, K.; Korabecny, J.; Bartolini, M.; Monti, B.; Lamba, D.; Caliandro, R.; Pesaresi, A.; Brazzolotto, X.; Gastellier, A.-J.; Nachon, F.; et al. Novel Tacrine-Tryptophan Hybrids: Multi-Target Directed Ligands as Potential Treatment for Alzheimer's Disease. *Eur J Med Chem* **2019**, *168*, 491–514, doi:10.1016/j.ejmech.2019.02.021.

S10. Viayna, E.; Coquelle, N.; Cieslikiewicz-Bouet, M.; Cisternas, P.; Oliva, C.A.; Sánchez-López, E.; Ettcheto, M.; Bartolini, M.; De Simone, A.; Ricchini, M.; et al. Discovery of a Potent Dual Inhibitor of Acetylcholinesterase and Butyrylcholinesterase with Antioxidant Activity That Alleviates Alzheimer-like Pathology in Old APP/PS1 Mice. *J Med Chem* **2021**, *64*, 812–839, doi:10.1021/acs.jmedchem.0c01775.
